# A Scoping Review on the Reported Evidence and Gaps of the Risk of Diabetes in Dyslipidemic Patients under Statin Therapy

**DOI:** 10.3390/clinpract12040060

**Published:** 2022-07-18

**Authors:** Jyotsna Needamangalam Balaji, Sreenidhi Prakash, Ashish Joshi, Krishna Mohan Surapaneni

**Affiliations:** 1 Panimalar Medical College Hospital & Research Institute, Varadharajapuram, Chennai 600-123, Tamil Nadu, India; jyotsna.nb@pmchri.ac.in (J.N.B.); sreenidhi.p@pmchri.ac.in (S.P.); 2 CUNY Graduate School of Public Health & Health Policy, New York, NY 10027, USA; ashish1875@gmail.com; 3 SMAART Population Health Informatics Intervention Center, Foundation of Healthcare Technologies Society, Panimalar Medical College Hospital & Research Institute, Varadharajapuram, Chennai 600-123, Tamil Nadu, India; 4 Departments of Biochemistry, Medical Education, Molecular Virology, Research, Clinical Skills & Simulation, Panimalar Medical College Hospital & Research Institute, Varadharajapuram, Chennai 600-123, Tamil Nadu, India

**Keywords:** statins, diabetes mellitus, dyslipidemia, insulin resistance, new-onset diabetes mellitus

## Abstract

With the increasing global burden of dyslipidemia over the past 30 years, it is estimated that more than 200 million people worldwide are under statin therapy. In India, roughly 25–30% of urban populations and 15–20% of rural populations have abnormal lipid levels. Statin, which is deemed to be the gold standard lipid-lowering agent, is the first treatment of choice for these patients. Although statins at one end are highly effective against dyslipidemiaand cardiovascular diseases, at the other end, they cause adverse effects including an increased risk of diabetes mellitus. The objective of this study was to understand the coexistence of diabetes and dyslipidemia in patients undergoing statin therapy. A scoping review was conducted with published articles selected from PubMed and Google Scholar. The obtained results were filtered based on inclusion/exclusion criteria. Our database search provided a total of 822 articles, of which 48 were selected for this review, with results concluding that statin users are potentially at a greater risk of developing diabetes mellitus compared with patients who are not using statins. Although many studies have been conducted to ascertain the onset of diabetes mellitus amongst statin users, the exact mechanism is not yet precisely established. Future studies are essential for identifying the exact cause of diabetes mellitus in statin users.

## 1. Introduction

Diabetes mellitus is a major public health concern with a current patient load of 537 million that is expected to rise exponentially by 2045. One in ten people worldwide are suffering from diabetes either diagnosed or undiagnosed. Every year, approximately 1.5 million deaths are associated with diabetic complications. The current estimation of the diabetic patient burden in India is 74 million, and that is expected to shoot up to 93 million by 2030 [1]. Drug-induced diabetes is found to be one of the leading causesof this rising trend [2]. According to the WHO, diabetes mellitus is a chronic condition with elevated levels of blood glucose that may lead to retinopathy, nephropathy, neuropathy and other cardiovascular diseases [3]. Increased blood glucose levels are mainly caused by (a) a deficiency in the production of insulin or (b) the decreased sensitivity of cells to insulin. Statins, a class of lipid-lowering drugs, are the gold standard treatment for dyslipidemia and other cardiovascular diseases. They competitively inhibit the key enzyme of cholesterol biosynthesis, HMG Co-A reductase.Statins can be broadly categorized as hydrophilic (pravastatin, rosuvastatin) or lipophilic (atorvastatin, fulvastatin, lovastatin, simvastatin, pitavastatin) [4].

The human body generally shows good endurance to statins, but at times it can be detrimental. The most common adverse drug reactions to statins are muscle-associated symptoms including myalgia, myopathyand elevated creatinine phosphokinase levels in the blood [5,6,7]. A few pieces of evidence also suggest that statins increase the risk of haemorrhagic stroke, impair memory and cognition, promote the formation of cataracts and affect kidneys, although these have not been demonstrated [6]. In many studies, statins have led to the progression of type 2 diabetes mellitus, insulin resistance, significant hyperglycaemia and its complications. The diabetogenic potential of statins is under study, and various mechanisms are being proposed, yet the exact cause is unknown. 

One of the main mechanisms through which statins cause diabetes mellitus is by increasing insulin resistance in peripheral tissues. Insulin resistance due to statins is caused by disturbances in the insulin signal transduction pathway. Even though insulin levels are normal in the blood, target cells fail to respond, leading to hyperglycaemia [4]. These statin-induced alterations in insulin signaling can be prevented by supplementing geranylgeranyl isoprenoid or promoting caspase 1 inhibition [8]. Statins also inhibit the biosynthesis of isoprenoid and down-regulate C/EBP α production, thereby causing insulin resistance [9]. Statins also enhance the endogenous production of glucose by activating key enzymes of gluconeogenesis such as PEPCK and glucose-6-phosphatase. This leads to hyperglycemia, which is a prominent feature of insulin resistance and diabetes mellitus [10]. 

Another proven mechanism is that statins decrease the blood cholesterol level by upregulating LDL receptor expression, increasing the uptake of LDL-C and downregulating the GLUT 4 transporter, affecting its protein expression. This will cause more cholesterol to enter the pancreatic beta cells, which causes damage to them. Thus, the pancreatic cells are dysregulated resulting in disrupted insulin secretion [6,11,12]. It is also to be noted that elevated triglyceride levels also increase insulin resistance [13]. Throughthese mechanisms, statins affect the utilization of glucose by peripheral tissues [4].

The calcium channels in pancreatic beta cells are disrupted due to cholesterol depletion, which results in decreased levels of insulin and ubiquinone which is a vital component of insulin signaling. Because of this, there is a decrease in beta cell ATP production, delay in insulin release and higher post-prandial glucose levels [9,14]. Statin users who have developed diabetes had both reduction in insulin secretion and increase in insulin resistance. Therefore, the principal mechanism by which statins alter glucose metabolism has not been proved with evidence [15,16]. The substrates required for protein prenylation are reduced by the statin-induced competitive inhibition of HMG CoA reductase. Reduced protein prenylation can elevate IL-1β. This pro-inflammatory cytokine can contribute to insulin resistance, which may be considered an element in the recent evidence linking statins to increased incidences of diabetes [8,17]. Other proposed mechanisms included are changes in ion channels, inflammatory/oxidative stress, modifications in adipocytes maturation, flux in leptin and adiponectin, and epigenetic and genetic changes that include alterations of micro-RNA [18].

Although multiple studies have been conducted and many mechanisms are proposed, the accurate reason for the onset of diabetes mellitus in this regard is not substantiated. To address the increasing crisis of diabetes linked with the use of statin, the need for further investigation arises. In this context, this scoping review was carried out to report on the current evidence and to recognize the gaps in the existing knowledge.

## 2. Materials & Methods

This scoping review included published research articles on statin-induced diabetes mellitus. A systematic search was conducted of the databases Google Scholar and PubMed. Selecting the literature, the data analysis, and the manuscript writing took place over a period of 20 days from 1 March 2022 until 20 March 2022. The PRISMA extension for scoping review was followed in organizing the structure of this review [19].

### 2.1. Stage 1: Sources of Information

A comprehensive search was performed in Google Scholar and PubMed. Published articles in the English language were selected.

### 2.2. Stage 2: Search Strategy

A broad search term, “statin-induced diabetes mellitus”, was used to obtain the primary results. To narrow the search results, “statin-induced insulin resistance” was used as the exact phrase, and “diabetic dyslipidemia” had to be at least one of the words. In PubMed, among the resulted articles, those which were published in the past 5 years from 2018–2022 were selected. These articles were further filtered based on the inclusion and exclusion criteria.

### 2.3. Stage 3: Process of Selection

**Figure 1:**
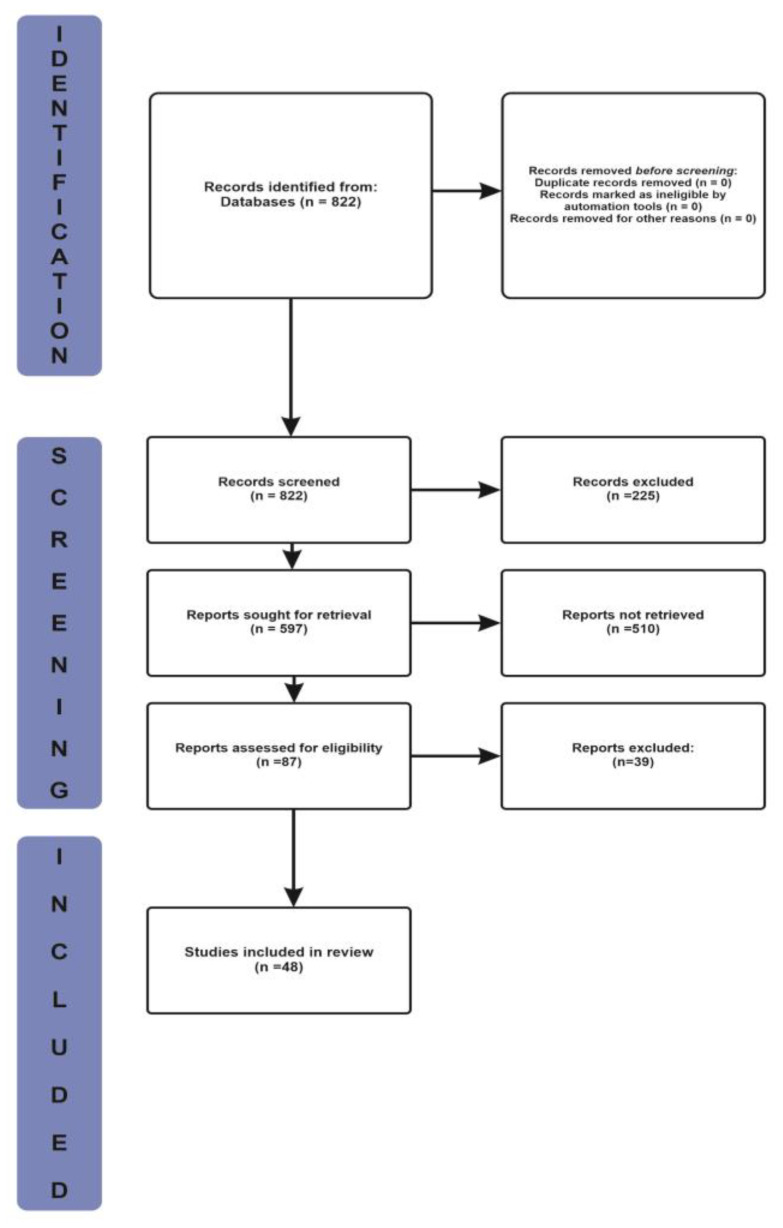
The Process of Selection.

The process of selection of articles includes three critical steps viz. Identification, Screening and Inclusion as shown in the Figure 1. The Identification step includes the information pertaining to, the records identified from the database and the records removed before screening. The Screening step includes the information pertaining to the records screened, records excluded, records sought for retrieval, records not retrieved, reports assessed for eligibility and records excluded. The Inclusion step shows the information pertaining to the studies included in this review.

### 2.4. Inclusion and Exclusion Criteria

The articles to be studied were selected based on the following inclusion criteria. Articles that did not satisfy the inclusion recommendations were excluded. The following table provides the inclusion and exclusion criteria for the selection of articles (Table 1).

### 2.5. Data Charting

A data chart was jointly developed with the variables that has to be extracted. This data was analyzed individually by the authors and data was segregated. This was later analyzed and discussed.

### 2.6. Data Items

The analyses of the selected sources were studied, and the data from these individual sources are orderly segregated into year of study, main objectives of study, study design, study population, population characteristics, type of statin used, comparison, follow-up, and the outcome of the study.

## 3. Results

### 3.1. Selection of Source of Evidence

A total of 822 results (416 from Google Scholar and 406 from PubMed) were obtained from the broad search term. Articles to be included in the review were selected from these 87 results based on the inclusion and exclusion criteria listed in Table 1. A total of 48articles were selected.

### 3.2. Characteristics and Results of Source of Evidence

Table 2 displays the various parameters taken into consideration for this scoping review. All the extracted data are represented under the mentioned variables.

### 3.3. Summary of Charted Data

#### Characteristics of Charted Data

The initial search provided 822 articles, of which 48 were selected for this study based on the inclusion and exclusion criteria mentioned earlier. Our analysis provided results that ascertained the development of diabetes among statin users. Most of the experimental studies advocating the above-mentioned results were published over the last 3 years (2019–2021), with the majority being published in 2019 (*n* = 5). A wide range of study designs was selected for this review such as (i) prospective cohort (*n* = 7); (ii) retrospective cohort (*n* = 5); (iii) prospective panel (*n* = 1); (iv) propensity-matched (*n* = 1); (v) randomized control trial (*n* = 1); (vi) clinical trial (*n* = 2); and (vii) meta-analysis (*n* = 1). Of the studies included for this review, the majority were conducted in European countries (*n* = 7), and the fewest were conducted in America (*n* = 5). Additionally, in the analysis of the durations of follow-up with statin therapy, most of the investigations took place over a period of 3 to 4 years (*n* = 3, each).

**Figure 2:**
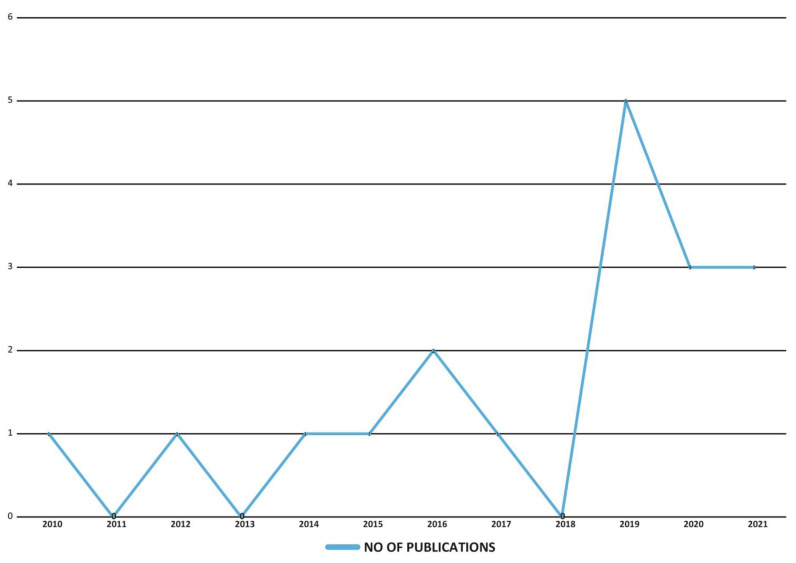
The number of publications included in the study.

Figure 2 shows the number of articles published each year from 2010 to 2021, those were selected for this review.

**Figure 3:**
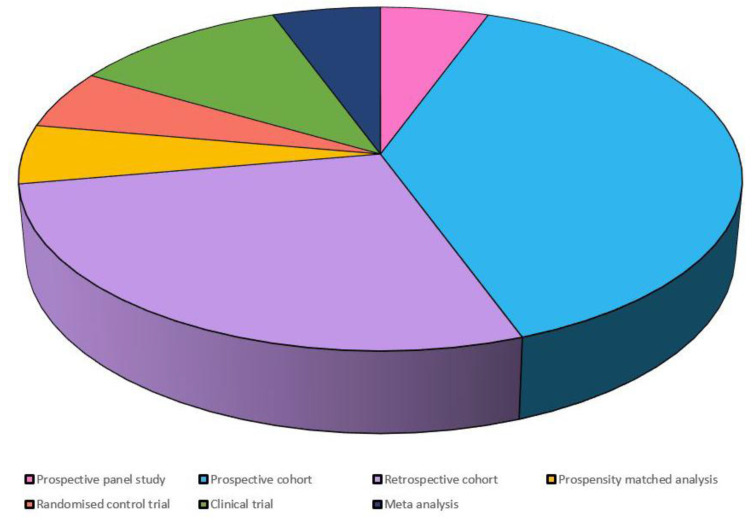
Thestudy designs analyzed.

Figure 3 depicts the number of articles included and analyzedby study design.

**Figure 4:**
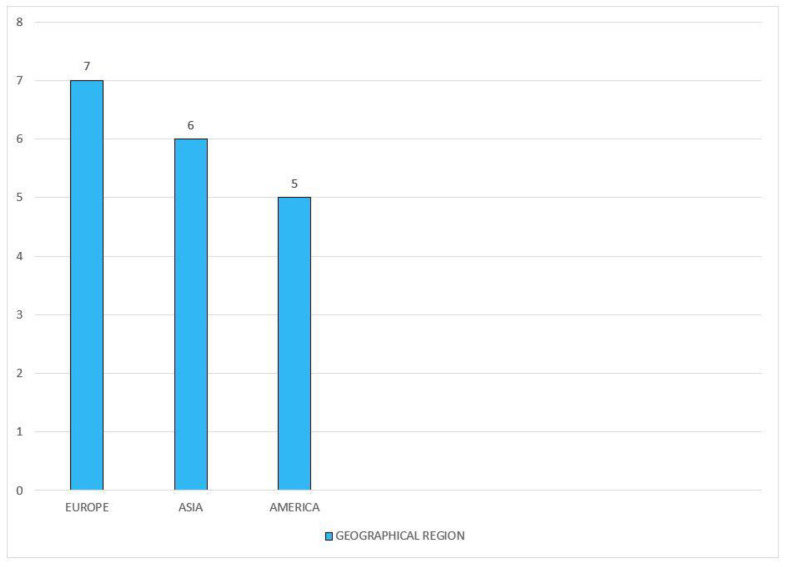
The geographical regions of the study publications.

The corresponding Figure 4 shows the numbers of publications in different geographical locations that were selected for this review.

**Figure 5:**
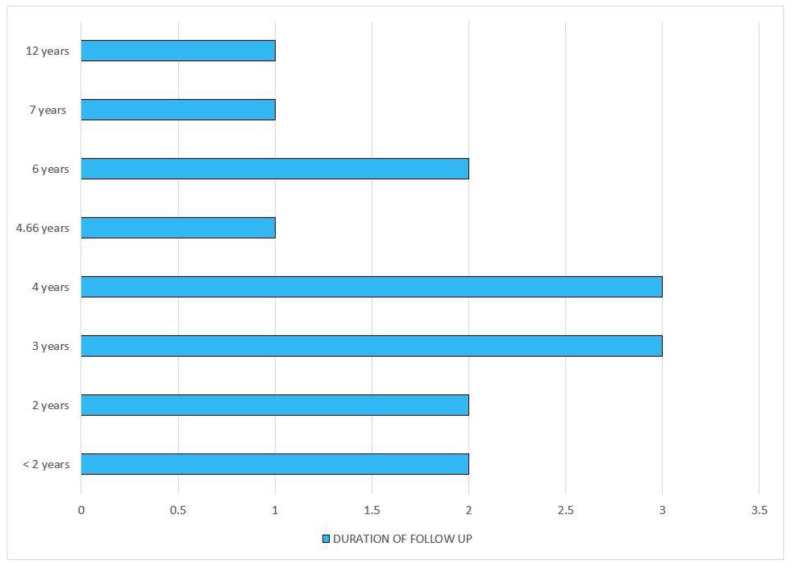
Duration of follow-up with statin therapy.

Figure 5 represents the duration of follow-up with statin therapy inthe studies included in this review.

From the data charted above, the following conclusionswere drawn.

The elevation in the glycemic parameters is evident among statin users compared to non-statin users, which signifies statin users may have nearly double the risk of developing diabetes than non-statin users [20,21,24]. One of the chronic complications of statin usage is diabetic neuropathy. An experimental study put forward the finding that when the statin wasadministered in combination with metformin, no significant neuropathy was manifested [22]. It has been found that statin users without dyslipidemia have increased insulin resistance as compared withstatin users with dyslipidemia [23]. From the data, it is also clear that increasing the consumption of statins will simultaneously increase the risk of developing diabetes [25]. It has also been stated that along with other risk factors, atherogenic dyslipidemia is an independent threat for diabetes [26]. Even though many studies have associated the risk factors withthe development of diabetes, a prospective, single-blinded, randomized trial contradicted this, stating that there was no significant relationship between the two [27]. In a trial that investigated the changes in the glycemic parameters among three groups of patients, with normoglycemia, impaired fasting glucose and impaired glucose tolerance, the effect of atorvastatin was found to be dose- and time-dependent. Increasing the intensity and duration of statin therapy was associated with significant changes in HbA1C levels in blood together with elevation in all other glycemic measurements [29,34]. A clinical trial conducted in the United States revealed that post-menopausal women under statin treatment had increased risk of developing diabetes [35]. In addition to this, epigenetics also has an influence over the diabetogenic effect of statins. This diabetogenicity of statins is enhanced by DNA methylation [33].

## 4. Discussion

We analyzed the association between diabetes mellitus and statin usage. Evidence suggeststhat lipophilic and hydrophilic statins may not have the same effects on glucosemetabolism. Increased doses of lipophilic statins lead to reduced insulin secretion, which may be attributed to the inhibition of HMG CoA/cytotoxicity. On analyzing the glycated hemoglobin, statin users were found to have elevated HbA1C levels that were particularly significant with atorvastatin compared withpravastatin [38,39].

The effect of statins on glucose metabolism varies from one statin to another [40]. Statins appear to have a dose dependent influence on development of diabetes [41]. Also, the risk of macrovascular complications is less in statin treatment without diabetes as compared to statin treatment with new onset of diabetes mellitus [25]. High-intensity statins are associated with a higher risk of diabetes than low-intensity statins [42]. The diabetogenicity of atorvastatinand simvastatin are higher than that ofpravastatin. Since pravastatin is hydrophilic, it has the least diabetogenic potential. Simvastatin and atorvastatin have the highest diabetogenic potential and affect glucose metabolism in a dose-dependent manner [28,29,43]. Atorvastatin, a lipid-soluble statin, exhibits its diabetogenic effects by decreasing insulin secretion due to HMG CoA inhibition. Pitavastatin improves insulin resistance andminimally impairs glucose metabolism [9]. Adiponectin shows a negative correlation between abdominal obesity and insulin resistance. Low levels may reduce insulin secretion and insulin sensitivity, whereas the risk of diabetes decreaseswith higher dosages. However, its exact role in this regard is not explored [39]. Studies have shown that patients with metabolic syndrome, who are at a greater risk of diabetes, can be safely treatedwith pitavastatin. The anti-inflammatory and anti-diabetic properties of pitavastatin are attributed to increasing adiponectin levels [27].

It was reported that statin therapy increases the risk of type 2 diabetes mellitus by 46%, worseninghyperglycemia, especially 2 h postprandial. [28].The incidence of diabetes was greater among those undergoing intense statin treatment [29]. Studies have shown that progression to diabetes is greater among statin users who possess risk factors including obesity, female gender, elderliness, Asian lineage, hypertension and hypertriglyceridemia as well as inpatients with borderline alterations in glycemic parameters and metabolic syndrome [6,26,39,44,45]. Age plays an important role in the association of T2DM withstatins. Statin-induced T2DM risk is potentially higher in younger populations and individuals with lower LDL-C levels [46].This remains as a controversy against statements proving that people with risk factors like older age are more prone to statin-induced diabetes.

As already stated, statin therapy significantly decreases LDL-C levels [6]. Individuals with low LDL-C levels also have a high vulnerability to diabetes mellitus [23]. The incidence of developing diabetes mellitus is linked with a greaterreduction in baseline LDL-C, potentially more than 30% reduction [39]. A bidirectional two-sample Mendelian randomized study was conducted that indicated that LDL-C response to statin is not likely to be influenced by T2DM liability. However, there is also some evidence to support the genetic relationship between statin-induced LDL-C response and T2DM [47].

A study treatment proved that inspite of the low levels of C-reactive protein, atorvastatin usage for more than 6 months caused reduced insulin sensitivity [48]. In dyslipidemia, insulin resistance causes the inflated flow of free fatty acids, which significantly increases the levels of triglycerides and LDL-C and decreases HDL-C levels in blood, which contributes to diabetes. This can be aggravated by higher levels of adipocytokines [49]. Even though the beneficial effects of statins outweigh the risk of developing adverse reactions, high-risk patients should be prescribed statins with caution to prevent the onset of diabetes mellitus [50,51]. The following factors are to be considered whenprescribing statins: dose and its diabetogenicity, patient factors, cardiovascular risk, diabetic risk, kidney function, liver function and other baseline metabolic parameters [40]. Adhering to these precautions will aid in achieving the goals of sustainable well-being and good health as well as reducing the mortality rates of noncommunicable diseases [52].

## 5. Conclusions

The current scoping review revealed a fair amount of literature regarding the causes and incidence of diabetes induced by statins. Statins promote a significant decrease in cholesterol levels, thus improving the condition of dyslipidemia. Although generally statins are safe, patients can develop adverse signs due to theirdiabetogenic effects. Various risk factors such as age, gender, obesity and conditions like impaired glucose tolerance and their impacts on diabetogenicity of statins have been discussed. To summarize all the evidence considered, the studies included for this review substantiate and emphasize the diabetogenic potential of statins.

## 6. Knowledge Gaps

Even though numerous studies have been carried out concerning the side effects of statins, the exact cause of the diabetogenicity remains unanswered. Certain proposed mechanisms like the decreased activity of small GTPase, the reduction in insulin secretion by the inactivation of leptins and the reduced peroxisomal proliferator-activated gamma receptors by arresting adipocytes differentiation that are expected to have an influence over the diabetogenicity of statins are yet to be explored deeply. Factors like age, gender, body weight, lipid levels in blood and other comorbid conditions should be individually evaluated tocategorize those as risk factors. The deleterious effects of smoking and alcohol consumption should be assessed when administering statins. Moreover, the relationships between the dose of statin administered and individual risk factors arenot quantitatively established. In addition to this, metformin, a drug given for type 2 diabetes mellitus, is known to reduce insulin resistance, but its efficiency under statin-induced diabetes is not yet explored. Moreover, many trials that have been conducted did not reveal which type of statin was used, andthis information is crucial in deciding the diabetogenicity of different statins.The studies conducted so far have also not revealed whether statins act as a precipitating factor only in patients with prediabetic conditions or discretely affect glucose metabolism.

## 7. Limitations

The design of this scoping review is subject to certain limitations. The published works of literature from only two databases were included for this study. Further, the selection of articles was restricted to those published in the English language. Publications were selected from the year 2009; older articles were not included as those may not provide appropriate details on the current knowledge of this issue due to advancements in diagnosis and treatment strategies. Lastly, as this study focused on the effects of statins on humans, all animal studies were excluded.

## 8. Directions for Future Research

Studies that focus on deciphering the exact mechanism producing the diabetogenic effects of statins should be conducted. The influence of patient-related factors such as age, gender and obesity should be analyzed in depth to find theircontributions to statin-induced diabetes mellitus. Furthermore, quantitative studies relating the dose of statin and its diabetogenic effects will help physicians in prescribing appropriate doses of statin. Understanding the efficacy of metformin under statin-induced diabetes will also aid in its treatment.

## Figures and Tables

**Table 1:** Criteria for the inclusion and exclusion of articles.

Criteria	Inclusion	Exclusion
Study design	Quantitative, qualitative and mixed-method designs.	Animal studies
Location	Low-, middle- and high-income countries	None
Year	2009–2022	Before 2009
Language	English	Any other language
Comorbidities	Dyslipidaemia	Pre-existing diabetes mellitus, pancreatitis and any others

**Table 2:** Extracted Data.

Reference	Year	Country	Main Objective	Study Design	Study Population	Population Characteristics	Statin Included	Comparison	Follow up Time	Outcome
[20]	2019	The Netherlands	To investigate the association of statin use with glycemic traits with incident type 2 diabetes	Prospective cohort study	9535 individuals	Age—64.3 (SD-10.1) years; 41% were men	Not specified	Non statin users	4 years	Statin users have increased serum fasting insulin and insulin resistance
[21]	2019	Korea	To find risk of diabetes mellitus associated with statin therapy	Prospective cohort study	1,034,982 individuals	Never statin users—Age 51.9 years, ever statin users—Age 55.5 years	Not specified	Never statin users	4 years	There are time- and dose-dependent associations of statin use with an increased risk of new-onset diabetes mellitus
[22]	2021	Korea	To evaluate the incidence of neuropathy among patients with T2DM associated with statin and metformin therapies	Prospective cohort study	2016—34,964 individuals; 2017—35,887 individuals	Age—30 to 70 years divided into 5 groups.	Not specified	Combined statin and metformin users	2 years	Statin users have increased risk of hyperglycemia, insulin resistance and type 2 diabetes mellitus
[23]	2020	Korea	To explore the relationship between baseline LDL—c and the risk of statin-induced insulin resistance	Propensity-matched analysis	2660 patients	Not mentioned	Not specified	None	2 years	Insulin resistance was higher in statin users without baseline dyslipidemia than inthose with dyslipidemia
[24]	2021	USA	To evaluate the progression of diabetes in diabetic patients after statin use	Retrospective cohort study	83,022 individuals	Mean age of 60.1, 94.9% were men	Not specified	Non-statin users with diabetes	12 years	The progression of diabetes was higher in statin users as compared to active comparators
[25]	2020	Italy	To find out the risk of macrovascular complications associated with type 2 diabetes induced by statins	Prospective cohort study	84,828 individuals	Age group—40 to 80 years	Not specified	Non-diabetic patients	6 years	The prognosis of statin-dependent type 2 diabetes is less adverse than that not induced by statins
[26]	2019	Greece	To explore the metabolic factors that influence the increased risk of diabetes mellitus in patients under statin therapy	Retrospective cohort study	1241 patients with dyslipidemia	Age group 46–64 years	Not specified	None	3 years	Atherogenic dyslipidemia is independently associated with NODM
[27]	2019	Korea	To identify the risk of developing diabetes in patients taking lowest and highest doses of pitavastatin	Single blinded randomized study	1044 patients	Age—30 to 79 years	Pitavastatin	Highest and lowest dose of Pitavastatin	3 years	There was no difference in the incidence of development of NODM between two groups
[28]	2015	Finland	To decipher the mechanism associated with statin induced diabetes mellitus	Prospective cohort study	8749 individuals	Age—45 to 73 years	Not specified	Never statin users	6 years	The factors contributing to increased diabetes risk are decreased insulin sensitivity and insulin secretion
[29]	2017	India	To analyze the effect of atorvastatin on the glycemic indexes of normoglycemic and prediabetic individuals	Prospective panel-based study	75 patients	Group A (N = 25), female (36%), age 53.95 years (SD = 6.9); Group B (N = 25), female 24%, age 51.05 years (7.1)	Atorvastatin	None	Up to 1.5 years	In normoglycemic patients, atorvastatin therapy induced glucose intolerance, and in prediabetic individuals, it led to diabetic progression
[30]	2010	UK	To establish the relationship between statinuse and onset of diabetes	Meta-analysis	91,140 participants	Not mentioned	Not specified	Not specified	4 years	There is a marginal increase in the risk of developing diabetes with the use of statins
[31]	2021	USA	To elucidate the physiological mechanism underlying the increased risk of diabetes mellitus in statin treatment	Clinical trial	75 participants	Median age 61 years, 37% women	Atorvastatin	None	10 weeks	High-intensity atorvastatin elevates blood glucose levels by increasing insulin resistance and decreasing insulin secretion
[32]	2016	Greece	To evaluate the risk of progression from prediabetes to diabetes in individuals under statin therapy	Retrospective cohort study	877 participants	Not mentioned	Not specified	Normoglycemic and prediabetic individuals	7 years	High-intensity statin treatment posed a higher risk of the development of diabetes in prediabetic individuals
[33]	2020	The Netherlands	To determine the role of epigenetics in statin-induced diabetes mellitus	Prospective cohort study	8270 participants	Age group; 35 to 75 years; 56% South Asian, 44% Caucasians	Not specified	None	Not specified	DNA methylation is associated with the diabetogenic potential of statins
[34]	2019	USA	To determine the risk of dysglycemia in patients under statin therapy	Retrospective cohort study	7064 participants	Not mentioned	Not specified	None	Not specified	Elevated HbA1c was observed in non-diabetic statin users.
[35]	2012	USA	To investigate the association between the use of statins and the onset of diabetes in postmenopausal women	Clinical trial	161,808 post-menopausal women	Age group—50 to 79 years	Not specified	None	3 years	Statin use is associated with increased risk of diabetes in postmenopausal women.
[36]	2014	UK	To evaluate the effects of statins on NODM	Prospective cohort study	2,016,094 individuals	Age group: 30 to 85 years	Not specified	Non statin users	Mean 4.66 years	Type 2 diabetes mellitus is proven to be associated with statin use.
[37]	2016	Korea	To investigate associations between statins and NODM in patients with ischemic heart disease	Retrospective cohort study	156,360 patients	Age group: ≥18 years	Variety of statins	None	Not mentioned	All statins are associated with increased risk of NODM in ischemic heart disease patients, with pravastatin having the lowest risk.

## Data Availability

The data that supports this study are available upon request from the corresponding author.
